# Supplementary material for: “Female genital schistosomiasis is a sexually transmitted disease”: Gaps in healthcare workers’ knowledge about female genital schistosomiasis in Tanzania
Source: PLOS Glob Public Health. 2022 Mar 23;2(3):e0000059. doi: 10.1371/journal.pgph.0000059 (PMC10021524; doi:10.1371/journal.pgph.0000059)
Supplement: S1 File — (DOC) [file pgph.0000059.s001.doc]

**FOCUS GROUP DISCUSSION GUIDE**

**Health workers**

**Duration: Approximately 2 hours.**

**INTRODUCTION**

We have selected you to represent your community as we value your experience, as health workers, regarding water use, cleanliness, schistosomiasis, and the effects that Covid-19 has had on water use as well as health services delivery, access, and utilization in this community. Data that will be generated from this discussion will help us in preparing and packaging interventions aimed at reducing the transmission of Female Genital Schistosomiasis (FGS) in this community.

**Discussion rules**

- Every participant will air their views with fear of any other participant.
- Giving each other time to speak. When one participant is speaking, others will be listening.
- Respecting the opinions/views of others. When we disagree in our arguments, but no one should ridicule the other.
- There is no right or wrong response. All opinions/views are equally important.

**I: HEALTH ISSUES IN THE COMMUNITY**

1. What are the health problems facing your community? Please, mention them.
2. Of those you have mentioned, which ones are the most important? Please, mention them.

For each of the health problem mention, probe the following:

- Prevalence?
- Severity?
- Disability?
- Morbidity?

1. Out of the health problems you have mentioned:
2. Which ones are associated with water contact?
3. Which ones are associated with sexual and reproductive health (among women and men?).
4. Have you ever heard about schistosomiasis? How is it called in Kisukuma and/or Kinyantuzu?

- Where did you hear it?
- From who did you hear?
- Is schistosomiasis a problem in your community? Why?
  - What is the prevalence?
  - What is the severity of the problem?

1. What is schistosomiasis?
2. What are the causes of schistosomiasis? (the sources of schistosomiasis)
3. How is schistosomiasis transmitted from one person to another?

Probe

- Behaviours that contribute to the transmission of schistosomiasis
  - Defecation in the water sources
  - Not using toilets/latrines (Open defecation)
  - Rice farming (in paddy fields)
  - Swimming in the water sources

1. In your opinion, what symptoms does a person infected with schistosomiasis display?

1. What parts of the human body are affected by schistosomiasis? (Stomach, liver, urinary bladder, sexual organs).
2. Which groups of people in your community are most affected by schistosomiasis?

- Children. Of which age? How are they affected?
- Men. Of which age? How are they affected?
- Women. Of which age? How are they affected?

*You have mentioned that women can also get infected with schistosomiasis.*

1. Have you ever heard about Female Genital Schistosomiasis?

Probe: If they have never heard about it, remind them what they have said in the previous question (in case they indicated that they have heard about it).

If they respond that they have heard about it:

1. What are the causes of Female Genital Schistosomiasis?
2. What symptoms does a woman who is infected with Female Genital Schistosomiasis display? Mention them.

Probe

- Blood in urine (haematuria)
- Abdominal and pelvic pain
- Increased vaginal discharge
- Pain with coitus (Dyspareunia)
- Post-coital bleeding
- Menstrual disorders
- Dysuria (pain or difficulty urinating)
- Genital lesions

1. Can a woman with Female Genital Schistosomiasis transmit it to another person?
   1. If yes, how?
   2. If no, why?
2. In your view, can a woman/girl infected with Female Genital Schistosomiasis infect her husband/sexual partner?
3. Which groups of women are at more risk of being infected with Female Genital Schistosomiasis?

Probe

- Older women. Why?
- Women in their reproductive age. Why?
- Girls. Why?

1. In your opinion, how do people associate Female Genital Schistosomiasis with other infections/diseases?

Probe: How do people associate Female genital Schistosomiasis with:

- HIV and AIDS
- Other sexual transmitted infections (e.g. [Gonorrhea](https://www.cdc.gov/std/gonorrhea/default.htm), [Syphilis](https://www.cdc.gov/std/syphilis/default.htm))
- Cervical cancer
- Ectopic pregnancy (or Extrauterine pregnancy)
- Miscarriage
- Infertility/sterility
- Other problems associated with fertility and pregnancy

1. In your opinion, what would men do if they would find out that their partners/wives are infected with Female Genital Schistosomiasis?
2. Based on your experience, what are the challenges that women face in accessing
3. Health services for Female Genital Schistosomiasis?
4. Other sexually transmitted infections (e.g. HIV/AIDS, [Gonorrhea](https://www.cdc.gov/std/gonorrhea/default.htm), and [Syphilis](https://www.cdc.gov/std/syphilis/default.htm))
5. Prenatal and antenatal services
6. In your opinion, how does the community perceive women/girls infected with Female Genital Schistosomiasis?

Probe

- How does the community regard/perceive a girl infected with Female Genital Schistosomiasis?
- How does the community regard/perceive an older woman infected with Female Genital Schistosomiasis?
- How does the community regard/perceive a married woman infected with Female Genital Schistosomiasis?
- Is there any kind of stigmatization against women/girls infected with Female Genital Schistosomiasis in this community?
  - Can you describe what form of stigmatization that is?
- Is there any kind of stigmatization against women/girls infected with sexually transmitted infections (e.g. HIV/AIDS, [Gonorrhea](https://www.cdc.gov/std/gonorrhea/default.htm), and [Syphilis](https://www.cdc.gov/std/syphilis/default.htm)) in this community?
  - Can you describe what form of stigmatization that is?

1. In your opinion, in order to reduce/decrease stigmatization against women infected with Female Genital Schistosomiasis

- What should the society/community do, considering that you are also members of the society/community?
- What should the government and other institutions do in this community?
- What should the government and other institutions do in the health sector?

1. In your opinion, what should be done in your community to encourage women and girls to seek for Female Genital Schistosomiasis health services?

Probe

- What should be done to encourage women to access health services for HIV/AIDS and other sexually transmitted infections ([Gonorrhea](https://www.cdc.gov/std/gonorrhea/default.htm) and [Syphilis](https://www.cdc.gov/std/syphilis/default.htm))?

**II: HEALTH SERVICES AND TREATMENT**

1. **Tell the group:** Think of a woman you know in your community (she can be a girl, sister, nephew, friend etc.) has some of the symptoms of Female Genital Schistosomiasis (for instance has blood in urine). Let as call her……… (Put a name). What is the first step that she will take? Why?

- Do health workers here have enough knowledge on Female genital Schistosomiasis?
- Can health workers know if the symptoms she displays are those of Female genital Schistosomiasis?
- Are there other infections/diseases that the health workers would think of first or prioritize instead of Female Genital Schistosomiasis?
- What are the health services available for Female Genital Schistosomiasis treatment?
- Do you think that health facilities have enough equipment to diagnose patients infected with Female Genital Schistosomiasis?
- If not, why?
- Probe: the availability of the medical equipment, health workers, incentives etc.

1. What are the challenges that face
2. Health workers providing services to women infected with Female Genital Schistosomiasis?
3. Health workers providing services to women infected with other sexually transmitted infections?

**III: COMMUNITY-BASED TEACHING INTERVENTION**

| Duration | 30 minutes |
| --- | --- |
| Moderator’s guide | Write the question on the flipchart  Draw the table on the flipchart for the second question for every group   | **Types of interventions** | **Why this intervention?** | **Who should deliver the intervention?** | **Anticipated challenges in implementing the intervention** | | --- | --- | --- | --- | |  |  |  |  | |

1. **CREATE GROUPS**: Request the participants to seat in pairs and discuss the following question:
   - What do you think should be done so that women and girls could get treatment services for Female Genital Schistosomiasis?

N.B. Ask every group to share their opinions/views and write them on the flipchart.

1. **SMALL GROUPS: Request groups to join other groups and discuss:**

- If the government or a non-governmental organization wants to support your community to fight against Female Genital Schistosomiasis, what kind of interventions could work (in this community?)
- Why do you think these interventions could work better?
- Who do you think should deliver these interventions?
- Are there challenges in implementing these interventions?

1. If the government or a non-governmental organization implements a community-based teaching intervention against Female Genital Schistosomiasis in your community (on raising awareness about Schistosomiasis, treatment and prevention of Schistosomiasis as well as women/girls’ seeking behaviour:

- Do you think this community-based teaching intervention can have positive impacts by improving women/girls’ awareness on health seeking behaviour and accessing treatment for Female Genital Schistosomiasis? **If yes, why? If no, why?**
- **If yes**, how should this community-based teaching intervention be run?
- **Probe:** Topics to be discussed, venue, teaching materials/equipment, time for the training, who should deliver the intervention etc.
- Do you think it is important to involve men in this community-based teaching intervention against Female Genital Schistosomiasis? If yes, why? If no, why?

1. In your opinion, do you think there will be any challenges in implementing this intervention?

- If yes, what are those challenges?
- In your view, how should those challenges be resolved?

1. Have you ever been trained on diagnosing Female Genital Schistosomiasis? If yes, when, where, who delivered the training (facilitator), and who funded the training?

**IV: EFFECTS OF COVID-19**

1. Have you ever heard about the covid-19 disease?

Probe:

- What is covid-19?
- How do you protect yourselves against covid-19?
- Where did you hear about covid-19?
- How has covid-19 affected:
  - The availability of water
  - Water use
  - Improvement of cleanliness

1. How has covid-19 affected health services delivery at the health facilities where you are working?

**THE END**

We have come to the end of our discussion. Do you have anything regarding the topic we have discussed that you would want to share with us? Or is there anyone with a question?

Thank you so much for your time and responses.
