# Supplementary material for: “Female genital schistosomiasis is a sexually transmitted disease”: Gaps in healthcare workers’ knowledge about female genital schistosomiasis in Tanzania
Source: PLOS Glob Public Health. 2022 Mar 23;2(3):e0000059. doi: 10.1371/journal.pgph.0000059 (PMC10021524; doi:10.1371/journal.pgph.0000059)
Supplement: S2 File — (DOC) [file pgph.0000059.s002.doc]

**INTERVIEW GUIDE FOR KEY INFORMANTS**

**Health workers**

**GENERAL UNDERSTANDING OF SCHISTOSOMIASIS**

1. What are the health problems facing your community? Please, mention them.
2. Of those you have mentioned, which ones are the most important? Please, mention them.

For each of the health problem mention, probe the following:

- Prevalence?
- Severity?
- Disability?
- Morbidity?

1. Out of the health problems you have mentioned:
2. Which ones are associated with water contact?
3. Which ones are associated with sexual and reproductive health (among women and men?).
4. Have you ever heard about schistosomiasis? How is it called in Kisukuma and/or Kinyantuzu?

- Where did you hear it?
- From who did you hear?
- Is schistosomiasis a problem in your community? Why?
  - What is the prevalence?
  - What is the severity of the problem?

1. What is schistosomiasis?
2. What are the causes of schistosomiasis? (the sources of schistosomiasis)
3. How is schistosomiasis transmitted from one person to another?

Probe

- Behaviours that contribute to the transmission of schistosomiasis
  - Defecation in the water sources
  - Not using toilets/latrines (Open defecation)
  - Rice farming (in paddy fields)
  - Swimming in the water sources

1. In your opinion, what symptoms does a person infected with schistosomiasis display?

1. What parts of the human body are affected by schistosomiasis? (Stomach, liver, urinary bladder, sexual organs).
2. Which groups of people in your community are most affected by schistosomiasis?

- Children. Of which age? How are they affected?
- Men. Of which age? How are they affected?
- Women. Of which age? How are they affected?

*You have mentioned that women can also get infected with schistosomiasis.*

1. Have you ever heard about Female Genital Schistosomiasis?

Probe: If they have never heard about it, remind them what they have said in the previous question (in case they indicated that they have heard about it).

If they respond that they have heard about it:

1. What are the causes of Female Genital Schistosomiasis?
2. What symptoms does a woman who is infected with Female Genital Schistosomiasis display? Mention them.

Probe

- Blood in urine (haematuria)
- Abdominal and pelvic pain
- Increased vaginal discharge
- Pain with coitus (Dyspareunia)
- Post-coital bleeding
- Menstrual disorders
- Dysuria (pain or difficulty urinating)
- Genital lesions

1. Can a woman with Female Genital Schistosomiasis transmit it to another person? Je,
   1. If yes, how?
   2. If no, why?
2. In your view, can a woman/girl infected with Female Genital Schistosomiasis infect her husband/sexual partner?
3. Which groups of women are at more risk of being infected with Female Genital Schistosomiasis?

Probe

- Older women. Why?
- Women in their reproductive age. Why?
- Girls. Why?

1. In your opinion, how do people associate Female Genital Schistosomiasis with other infections/diseases?

Probe: How do people associate Female genital Schistosomiasis with:

- HIV and AIDS
- Other sexual transmitted infections (e.g. [Gonorrhea](https://www.cdc.gov/std/gonorrhea/default.htm), [Syphilis](https://www.cdc.gov/std/syphilis/default.htm))
- Cervical cancer
- Ectopic pregnancy (or Extrauterine pregnancy)
- Miscarriage
- Infertility/sterility
- Other problems associated with fertility and pregnancy

1. In your opinion, what would men do if they would find out that their partners/wives are infected with Female Genital Schistosomiasis?
2. Based on your experience, what are the challenges that women face in accessing
3. Health services for Female Genital Schistosomiasis?
4. Other sexually transmitted infections (e.g. HIV/AIDS, [Gonorrhea](https://www.cdc.gov/std/gonorrhea/default.htm), and [Syphilis](https://www.cdc.gov/std/syphilis/default.htm))
5. Prenatal and antenatal services
6. In your opinion, how does the community perceive women/girls infected with Female Genital Schistosomiasis?

Probe

- How does the community regard/perceive a girl infected with Female Genital Schistosomiasis?
- How does the community regard/perceive an older woman infected with Female Genital Schistosomiasis?
- How does the community regard/perceive a married woman infected with Female Genital Schistosomiasis?
- Is there any kind of stigmatization against women/girls infected with Female Genital Schistosomiasis in this community?
  - Can you describe what form of stigmatization that is?
- Is there any kind of stigmatization against women/girls infected with sexually transmitted infections (e.g. HIV/AIDS, [Gonorrhea](https://www.cdc.gov/std/gonorrhea/default.htm), and [Syphilis](https://www.cdc.gov/std/syphilis/default.htm)) in this community?
  - Can you describe what form of stigmatization that is?

1. In your opinion, in order to reduce/decrease stigmatization against women infected with Female Genital Schistosomiasis

- What should the society/community do, considering that you are also members of the society/community?
- What should the government and other institutions do in this community?
- What should the government and other institutions do in the health sector?

1. In your opinion, what should be done in your community to encourage women and girls to seek for Female Genital Schistosomiasis health services?

Probe

- What should be done to encourage women to access health services for HIV/AIDS and other sexually transmitted infections ([Gonorrhea](https://www.cdc.gov/std/gonorrhea/default.htm) and [Syphilis](https://www.cdc.gov/std/syphilis/default.htm))?

1. How big is the problem of Female Genital Schistosomiasis compared to other health problems in the community where you are serving?
2. From your experience, is there a woman/girl in your family or a neighbor that has ever been infected with Female Genital Schistosomiasis? If yes, please explain to me about this scenario.
3. In this community, when a person has symptoms of Schistosomiasis where do they go to seek for treatment services?

Probe

- To the traditional healers?
- To the hospital (biomedical facility)?
- To the private retail drug shops?
- To look for self-medication?

1. Do you think that health facilities have enough equipment to diagnose patients suspected of being infected with Female Genital Schistosomiasis?

Probe

- Are there enough health workers?
- Are the medical equipment for gynecological examination available?
- Are the lab services available, good, and functioning?
- Are there lab technicians? Are they enough?
- Are medications available?

1. In your opinion which age groups of women come to seek for Female Genital Schistosomiasis treatment, HIV/AIDS, and other sexually transmitted infections?

Probe:

- Older women
- Women of reproductive age
- Young women and girls
- Female students/pupils.

1. (a) What should be done in order to raise more awareness and knowledge about Female Genital Schistosomiasis?

Probe

- Who should participate in the awareness raising campaigns?

(b) What should be done in order to increase the availability of health services for the treatment of Female Genital Schistosomiasis?

1. If the government or a non-governmental organization implements a community-based teaching intervention against Female Genital Schistosomiasis in your community (on raising awareness about Schistosomiasis, treatment and prevention of Schistosomiasis as well as women/girls’ seeking behaviour:

- Do you think this community-based teaching intervention can have positive impacts by improving women/girls’ awareness on health seeking behavior and accessing treatment for Female Genital Schistosomiasis? **If yes, why? If no, why?**
- **If yes**, how should this community-based teaching intervention be run?
- **Probe:** Topics to be discussed, venue, teaching materials/equipment, time for the training, who should deliver the intervention etc.
- Do you think it is important to involve men in this community-based teaching intervention against Female Genital Schistosomiasis? If yes, why? If no, why?

1. In your opinion, do you think there will be any challenges in implementing this intervention?

- If yes, what are those challenges?
- In your view, how should those challenges be resolved?

1. Have you ever been trained on diagnosing Female Genital Schistosomiasis? If yes, when, where, who delivered the training (facilitator), and who funded the training?
2. Have you ever heard about the covid-19 disease?

Probe:

- What is covid-19?
- How do you protect yourselves against covid-19?
- Where did you hear about covid-19?
- How has covid-19 affected:
  - The availability of water
  - Water use
  - Improvement of cleanliness

1. How has covid-19 affected health services delivery at the health facilities where you are working?

**THE END**

We have come to the end of our discussion. Do you have anything regarding the topic we have discussed that you would want to share with us? Or is there anyone with a question?

Thank you so much for your time and responses.
