# Supplementary material for: “Female genital schistosomiasis is a sexually transmitted disease”: Gaps in healthcare workers’ knowledge about female genital schistosomiasis in Tanzania
Source: PLOS Glob Public Health. 2022 Mar 23;2(3):e0000059. doi: 10.1371/journal.pgph.0000059 (PMC10021524; doi:10.1371/journal.pgph.0000059)
Supplement: Attachment — Submitted filename: Response to Reviewer Comments.docx [file pgph.0000059.s003.docx]

Response to Reviewer Comments

Dear Editor

Find point-to-point responses

| **S/no** | **Editorial comments** | **Response to comments** |
| --- | --- | --- |
| 1 | We suggest you thoroughly copyedit your manuscript for language usage, spelling, and grammar. If you do not know anyone who can help you do this, you may wish to consider employing a professional scientific editing service. | - We request Prof. Jenifer Downs who is a native English speaker from Cornell Medical School in New York to read the manuscript and help on English grammar and structures. See attached document with track changes |
| 2 | The name of the colleague or the details of the professional service that edited your manuscript | - Prof. Jenifer Downs form Cornell University in New York |
| 3 | A copy of your manuscript showing your changes by either highlighting them or using track changes (uploaded as a *supporting information* file) | - Copy submitted |
| 4 | A clean copy of the edited manuscript (uploaded as the new *manuscript* file) | - Attached |
| 3 | Please include a copy of the interview guide used in the study, in both the original language and English, as Supporting Information, or include a citation if it has been published previously. | - Provided in Kiswahili and English |
| 4 | Please provide separate figure files in .tif or .eps format only. | - Figures are provided in TIF |
| 5 | 4. In the online submission form, you indicated that "Qualitative data collected in this study contains information which can make participants identifiable. Thus, the data cannot be shared publicly."  All PLOS journals now require all data underlying the findings described in their manuscript to be freely available to other researchers, either 1. In a public repository, 2. Within the manuscript itself, or 3. Uploaded as supplementary information. This policy applies to all data except where public deposition would breach compliance with the protocol approved by your research ethics board. If your data cannot be made publicly available for ethical or legal reasons (e.g., public availability would compromise patient privacy), please explain your reasons by return email and your exemption request will be escalated to the editor for approval. Your exemption request will be handled independently and will not hold up the peer review process, but will need to be resolved should your manuscript be accepted for publication. One of the Editorial team will then be in touch if there are any issues. | - The section has been revised to meet the PLOS requirements |
| 6 | Please amend your detailed Financial Disclosure statement. This is published with the article, therefore should be completed in full sentences and contain the exact wording you wish to be published.  i). State what role the funders took in the study. If the funders had no role in your study, please state: “The funders had no role in study design, data collection and analysis, decision to publish, or preparation of the manuscript.” | - All the necessary information has been provided as per recommendation |
| 7 | Please ensure that the funders and grant numbers match between the Financial Disclosure field and the Funding Information tab in your submission form. Note that the funders must be provided in the same order in both places as well. Currently, the funders listed in the Funding Information section do not match the funders in the Financial Disclosure statement. | - The provided information are correct |
| 8 | Please provide us with a direct link to the base layer of the map used in Figure 1 and 2 and ensure this location is also included in the figure legend. | - Removed all the figures |
| 9 | Please amend your detailed Financial Disclosure statement. This is published with the article, therefore should be completed in full sentences and contain the exact wording you wish to be published. | - The provided statement is correct |
| 10 | iState what role the funders took in the study. If the funders had no role in your study, please state: “The funders had no role in study design, data collection and analysis, decision to publish, or preparation of the manuscript.” | - The statement is added |
| Reviewer comments | | |
| 1 | - Reviewer comments in track changes in the manuscript | - All comments provided in track changes have been attend (see the manuscript with track changes) |
